# Strengths within the Community Perceived by Older Adults Living Alone in a Semi-Mountainous Rural Region: A Qualitative Study

**DOI:** 10.3390/nursrep14020064

**Published:** 2024-04-02

**Authors:** Ai Nakai, Ikuharu Morioka

**Affiliations:** 1 School of Nursing, University of Kochi, Kochi 781-8515, Japan; 2 Graduate School of Health and Nursing Science, Wakayama Medical University, Wakayama 641-0011, Japan; moriokai@wakayama-med.ac.jp

**Keywords:** rural area, community, strengths, older adults, living alone, qualitative research

## Abstract

It is recommended that health promotion activities in the community focus on residents’ strengths. Hence, this study explored the community strengths perceived by older adults living alone in a semi-mountainous rural region of Japan. A qualitative, descriptive approach was used. Content analysis was performed using data obtained through face-to-face interviews. Interview data were coded; codes were classified based on similarity to create subcategories and categories. The strengths within the community, as perceived by older adults living alone in a semi-mountainous rural region, were revealed in four categories related to ten subcategories: “loose connections with others”, “active community participation”, “close relationships with community professionals”, and “familiarity with the living environment”. Strengths within the community perceived by older adults living alone in a semi-mountainous rural region were cultivated in an environment formed by their past lives. Utilizing these resources may help support community-based societies in semi-mountainous rural regions where depopulation and aging are expected to continue in the future. This study was not registered.

## 1. Introduction

The number of older single-person households in Japan is rapidly increasing, and the increase in the number of older adults living alone is particularly serious in semi-mountainous rural regions [1,2]. In these regions, social resources are scarce due to geographical characteristics, and because of the fragile mental and physical functions of older adults, those living alone are particularly vulnerable to physical, psychological, social, and environmental influences [3], which can easily make daily life in the community difficult and lead to emigration. Therefore, health promotion efforts in these regions are needed to enable residents to live in a familiar place until the end of their lives [4].

Health promotion activities in the community are important, and activities focusing on residents’ strengths are recommended [5]. For older adults living alone in semi-mountainous rural regions to continue living in familiar areas, it is expedient to provide support that makes use of the strengths within the community. Accordingly, it is necessary to first understand the strengths within the community as perceived by older adults living alone in these regions. Accordingly, this study clarified the strengths within the community as perceived by older adults living alone in a semi-mountainous rural region.

### Conceptual Framework: Strengths within the Community

An earlier literature review attempted to conceptualize the strengths within the communities involved in public health nursing [6]. The strengths within a community perceived by older adults are those that are subjectively, empirically, and positively perceived by them in their daily lives in the community. Therefore, in this study, based on previous literature [7], strengths within the community were defined as the social, cultural, and economic resources that positively impacted the participant’s life as judged by their own experiences. These strengths included the infrastructure and the general characteristics of a remote community, such as the individuals living there and their personality or other characteristics, social cohesion, social networks, and their history as a community.

Studies have reported strengths in the daily life of older adults living alone in the community [8] and those related to environmental aspects of older adults in the community [9]. Some have discussed individual strengths, such as mastery, sense-making, and openness to one’s vulnerability among older adults receiving care [10], and others have discussed three domains that seem to relate to individual strengths, which may also relate to the community, such as social policy and accessibility of care [11]. Enhancing the strengths of communities effectively promotes physical health, fulfilling social relationships and inner strength [12]. However, to date, no studies have examined strengths within the community in the context of older adults living alone in semi-mountainous rural areas.

To solve food access problems in semi-mountainous rural regions, environmental factors are more likely to be important than other factors in the environment of older adults living alone, as considered from multiple perspectives [7]. Earlier reports on these regions primarily focused on the geographical characteristics of the regions, the inconvenience of the physical environment, and poor living conditions of older adults, such as inadequate access to food due to topography [13], poor quality of life [14], and the importance of personal empowerment of late-stage older adults [15]. However, no studies have focused on the strength of a community as perceived by older adults living alone.

Based on earlier reports, the strength within the community in these regions appears to be that the local residents maintain regional connections and help each other even as the population continues to decline [6]. Even if access is poor due to the nature of the region, they effectively use the limited social resources and interact with family and local professionals [9]. Exploring the strengths within the community perceived by older adults living alone in these regions is important for supporting a community-symbiotic society in these regions in a super-aging society [4].

The first step in health promotion in the community is to understand the strengths within the community. Thus, a research framework was developed to clarify the strengths within the community as perceived by older adults living alone in semi-mountainous regions.

## 2. Materials and Methods

### 2.1. Study Design

This was a qualitative descriptive study using content analysis.

### 2.2. Target Population and Participants

In this study, a semi-mountainous rural area was defined as one with poor geographical conditions, represented by few flat arable lands, ranging from the periphery of the plains to mountainous areas [16]. The target population in this study was older adults living in a semi-mountainous rural area who could independently conduct activities of daily living (ADLs). The selection criteria for participants were those who were 65 years of age or older, lived alone at home, and could engage in normal daily conversations. Exclusion criteria were difficulty in communication and receiving home medical care.

Town A, the target area, is located in a semi-mountainous rural area of Japan; mountainous areas account for 96% of the town, whose population is composed of 45% older persons. Purposive sampling was used to recruit participants. We explained the purpose and procedures of the study to the representative in charge of the Community Welfare Division of the town. After obtaining informed consent, we asked the person in charge to introduce participants to the study. Ten individuals were introduced to the study.

### 2.3. Data Collection

To develop an interview guide, elements of strength within the community were extracted from the literature [6,10,11,15,17]. Questions covered age; marital status; number of years of residence; participation in meetings held by residents’ association organizations or the regional government; interaction and mutual help among residents; attachment to the local culture, climate, and natural environment; and the positive aspects of the region. The questions used in the interview guide were discussed among researchers involved in qualitative research. Additionally, we conducted a community assessment of the target area and evaluated its characteristics.

Data were collected through individual interviews with participants who had signed the consent form. A semi-structured interview method was used to encourage the participants to speak freely. The interviews were conducted in a room where privacy was ensured on a day when meetings involving older adults in the community were held. The first author conducted the interviews, which lasted approximately 30–40 min per person. The interviews were recorded using an IC recorder with the consent of the participants, and a verbatim transcript was made. Data collection was conducted in November 2021.

### 2.4. Analysis

Data were analyzed using inductive content analysis [18,19]. Content analysis extracts data from various types of interviews, observation protocols, diaries, websites, and medical records [20] and determines both the depth and meaning of participants’ utterances or recordings [19]. Inductive content analysis is conducted to derive latent concepts from the data [21] when earlier studies dealing with the phenomenon are lacking or when the phenomenon is fragmented [18]. In this study, we used inductive content analysis because we aimed to clarify the strengths within the community perceived by the older adults.

First, verbatim transcripts of the interview data were created. All authors read the content of the verbatim transcript carefully, organized the descriptions such that the contents were not inaccurate, and excluded incomprehensible content or unclear meanings of the written content from the analysis process. Descriptions were labeled based on their similarity, and a code was created with a unit in the semantic expressions of the interview data such that the meaning of the described content would not be impaired [19]. Additionally, the codes were classified based on code similarity, and subcategories were created with increased abstraction. Finally, we identified subcategories based on their similarities and generated categories with increased abstraction [19]. Table 1 presents the analysis process using an example dataset.

To ensure the rigor of the analysis, several factors were tightly controlled. To increase transferability, we described and recorded the process in detail under the supervision of experts familiar with qualitative research. To increase credibility, we returned the original data several times to confirm the content and shared them among the researchers through peer briefings. Transcription and audio recordings were used to enhance reliability. To increase confirmability in all analytical processes, the two researchers cross-checked the original data and repeatedly reviewed and refined the participants’ narratives and results.

### 2.5. Ethical Considerations

The participants had already given their consent to participate based on an explanation from the person in charge of Town A. However, on the day of the interview, the first author explained the following points in an easy-to-understand manner using both verbal and written forms: the purposes and methods of the study, that participation was voluntary, nonparticipation or withdrawal would not result in any disadvantages such as medical and social services received in daily life, privacy would be adequately protected, data would not be used for anything other than this study, anonymity would be maintained and strictly managed, questions were allowed to be asked freely, it was not necessary to answer questions that the participants did not want to answer, and the results of this study may be presented at oral sessions or in academic journals. After the participants understood the content of the explanation, they were asked to provide informed consent to participate in the study. During the interviews, we attempted to make the explanations easy to understand and were careful not to be inductive or coercive.

This study was approved by the Wakayama Medical University Ethics Review Committee (approval number 3254).

## 3. Results

### 3.1. Profiles of Participants

Nine participants agreed to participate in this study. The ages of the participants ranged from 75 to 96 years, and five were in their 80s (Table 2). Eight participants were women. All participants had resided in Town A for over 25 years and were married. Four had family members living in the same prefecture. Six participants engaged in social activities more than once per week. All participants did not have long-term insurance and eight participants had hypertension.

### 3.2. Analysis Results

Strengths within the community, as perceived by older adults living alone in a semi-mountainous rural region, emerged in four categories related to ten subcategories (Table 3). The categories were “loose connections with others”, “active community participation”, “close relationships with community professionals”, and “familiarity with the living environment”.

#### 3.2.1. Categories: Loose Connections with Others

The participants had casual relationships with their neighbors and were able to help each other without hesitation. We were able to extract the concept of sustained loose connections in the community. Additionally, connections with relatives living in the same prefecture were maintained.

Subcategories: Casual relationships with neighbors

“I don’t socialize with my neighbors every day. Well, I am having an easy time”.

I-96

“I often talk to someone. I’ve seldom been stuck at home”.

WD4

Subcategories: Help each other without hesitation

“I don’t hesitate to ask a neighbor to give me a ride. We have such kinds of mutual help”.

WC51

“I have bad legs, so I go out once every two months with a friend giving me a ride”.

WE2

Subcategories: Sustaining loose connections in the community

“I don’t go to salons so often, but I know everyone in the community. My neighbors often come to visit me”.

WI36

Subcategories: Connections with relatives

“I frequently communicate with my siblings by phone”.

WA93

“Because of my illness and COVID-19 crisis, my children won’t come to me at all. For the time being, I just talk on the phone”.

WC25

#### 3.2.2. Categories: Active Community Participation

The participants played roles in the community, such as promoting dietary habits and providing meals at the salons. Even if they did not play a role, they participated in community activities with the support of professionals in the community, such as public health nurses.

Subcategories: A role in the community

“The residents around here are younger than I, but they are already in their 70s and 80s. Well, people around the age of 70 are very cooperative with staff promoting dietary habits etc. That’s why life in this community is possible”.

WG84

“I am the only one who drives a car among the people using this salon. When someone becomes a representative, he or she would have to go to the community office several times a year and attend meetings. I’m already old, but I can drive a car, so I am a representative”.

WF8

Subcategories: Participation in community activities

“Community volunteers and public health nurses always advertise exercises and events. They frequently take me to the venue so that I can attend such events”.

WA73

“I come to the salon not every time, but when I can. Yes, this is because the community professionals always invite me”.

WB23

#### 3.2.3. Categories: Close Relationships with Community Professionals

There was considerable support available from community professionals, such as public health nurses and volunteers watching older adults’ households, such as guiding residents to events and encouraging them to participate. We found a close relationship between older adult residents and community professionals who could be consulted if necessary.

Subcategories: Strong support from community professionals

“Public health nurses often come to see me. The town officers and community professionals also visit me and take care of me”.

WA68

“The town officers came to see me the other day. Six staff members are engaged in a nursing home. They frequently guide me in my daily life. That’s why I happily live every day”.

WI29

Subcategories: Face-to-face relationship with community professionals

“I am acquainted with the people in the town office, and I often have conversations with them”.

WC45

“The person in charge of welfare visits me from time to time, so I talk with them”.

WI57

#### 3.2.4. Categories: Familiarity with the Living Environment

Although there were no supermarkets nearby, the participants obtained vegetables and other ingredients from mobile vendors who sold them near their homes or by growing them themselves. We found that ingredients were available through mobile vendors or grown at home. Despite the inconveniences, the participants were used to living in the area and felt comfortable.

Subcategories: Acquisition of ingredients through mobile vendors or home-growing

“I acquire everything through mobile vendors and grow vegetables in-house. I’m enjoying cooking and eating meals at the salon”.

WE4

Subcategories: Comfortable feeling for place

“It seems to be difficult to live here without a car. But I have lived here for a long time, so it’s already natural”.

WG101

## 4. Discussion

The participants in this study had resided in a semi-mountainous rural region for over 25 years and identified strengths within the community. Although less than half of them had relatives living in the same prefecture, they lived alone while maintaining relationships with relatives.

The strengths within the community perceived by older adults living alone in a semi-mountainous rural region were revealed in four categories. They understood the strengths within the community in relation to local residents, community professionals, the community, and the environment.

Regarding the category “loose connections with others”, the participants perceived the connection with neighboring residents, who were neither too close nor too far away, as a strength. Earlier studies reported various regional connections [9]. However, the loose connection perceived by older adults living alone in semi-mountainous areas has not been previously described, and this is considered to be a characteristic of such rural dwellings. As loose connections with neighboring residents were maintained, residents seemed to feel a sense of solidarity, even when living alone, and experienced mental stability.

“Close relationships with community professionals”, based on the communication with public health nurses and volunteers watching older-adult households, were recognized as a strength. The professionals in this area seemed to actively encourage residents to participate in local activities, such as salons, and to promote their social activities. Our previous research has also reported a proactive approach of community professionals to help undernourished residents [22]. In addition to positive relationships with relatives, strengthening relationships with professionals appeared to be a form of support for older adults in the community [11].

Participants regarded playing specific roles through “active community participation” as a strength. Older adults living alone in semi-mountainous rural regions tend to become confined as they age, and their connections with local residents may weaken. Thus, community professionals seemed to prevent the isolation of local residents by consciously calling them to participate in community activities. Older adults living in mountainous areas have a higher likelihood of being mentally healthy [14]. Providing them with opportunities to participate in social and leisure activities in the community might positively affect their quality of life [23]. Active participation in community activities, where the number of older adults living alone is increasing, may enable older adults to feel fulfilled and create fun.

Participants regarded “familiarity with the living environment” in their lives for over 25 years as a strength. In semi-mountainous rural regions, access to going out is often challenging; hence, it is difficult to consult a doctor or go shopping. Consequently, transportation is often developed by city governments to promote going out among local residents; however, this has not been done in the target region. Regarding the acquisition of ingredients, they seemed to be acquired through home growing, mobile vendors, and support from participants’ families. In earlier studies, because support was necessary for older adults living alone in the community in need of long-term care, support for shopping and social services was developed, and family members and friends assisted with their shopping [8]. Attachment to the region may explain why the participants in this study adapted to the environment in the semi-mountainous rural region where depopulation progressed and food stores decreased [6].

Although this study was conducted in a semi-mountainous rural region, the participants perceived cooperation among residents, mutual support, and partnerships with community professionals during aging and depopulation as strengths. As such, utilizing these resources may help support community-based societies.

To create a future community-based society [4], the strengths revealed in this study must be applied. For example, older adults living alone must participate in social activities and play a role in the community to find their own purpose in life, live life their own way, and prevent long-term care. Moreover, partnerships with community professionals may allow them to respond flexibly to the support needs of older adults living alone.

Community health workers, volunteers, and other supporters in semi-mountainous rural areas, where depopulation is expected to continue and the number of older adults living alone is expected to increase [4], are also aging. Therefore, there is a concern regarding the shortage of human resources. To effectively use the limited resources in a familiar living environment, it is desirable to have a system that allows nurses working at local clinics to participate in community activities.

### Challenges and Limitations of This Study

As this study analyzed data obtained from nine older adults living alone in a semi-mountainous rural region, the concepts obtained in this study may not have reached saturation, and it is not clear if the categories were independently extracted. Additionally, most participants were women, which might present selection bias. However, even with these limitations, we believe that the results of this study will help inform further research with a larger sample in other regions. The findings of this study have elements useful for healthcare professionals collaborating with residents in the community to provide support. Such support could help older adults living alone in community-based societies in semi-mountainous rural regions to live longer in their own way, given depopulation and aging, which are expected to continue in the future.

## 5. Conclusions

Strengths within the community, as perceived by older adults living alone in mountainous rural regions, consisted of four categories. These resources have the possibility to facilitate seamless, long-term care from the hospital to the community in such regions. The findings of this study will help community professionals collaborate with local residents to support older adults living alone so that they can live in their own way as long as they want and will help contribute to strengthening collaboration among healthcare providers such as nurses, physicians, community health volunteers, and caregivers.

## Figures and Tables

**Table 1:** Example of process for emerged categories in the analysis.

A Unit in Semantic Expressions of the Interview Data	Code	Subcategories	Categories
We live close to each other’s houses. I know him/her. He/she knows me. If he/she would not have seen me for a while, he/she would say something like “It’s been a while” or “I haven’t seen you. Are you not feeling well?” Most people live alone, so everyone takes care of them. If I dry my laundry or futon outside forever, they would call me. So, I feel like I’m already indebted to the local people. F-55	I live alone most of the time, so everyone takes care of me and talks to me.	Casual relationship with neighbors	Loose connections with others

**Table 2:** Characteristics of participants (*n* = 9).

Demographics	Categories	*n*	%
Age (years)	90–100	2	22.2
(Range 75–96)	80–89	5	55.6
	70–79	2	22.2
Gender	Male	1	11.1
	Female	8	88.9
Length of residence (years)	71–100	3	33.3
(Range 25–96)	51–70	3	33.3
	25–50	3	33.3
Marriage status	Bereavement	8	88.9
	Divorce	1	11.1
Participation in a social activity	Every week	6	66.7
	Several times a month	3	33.3

**Table 3:** Strengths within the community perceived by older adults living alone in a semi-mountainous rural region.

Categories	Subcategories
Loose connections with others	Casual relationships with neighbors
	Helping each other without hesitation
	Sustaining loose connections in the community
	Connections with relatives
Active community participation	A role in the community
	Participation in community activities
Close relationships with community professionals	Strong support from community professionals
	Face-to-face relationship with community professionals
Familiarity with the living environment	Acquisition of ingredients through mobile vendors or grown at home
	Comfortable feeling for place

## Data Availability

The datasets used and/or analyzed in the current study are available upon reasonable request to the corresponding author.

## Public Involvement Statement

There was no public involvement in any aspect of this research.

## Guidelines and Standards Statement

This manuscript was drafted following the Standards for Reporting Qualitative Research (SRQR) for qualitative research.
